# Succinic Acid Production from Monosaccharides and Woody and Herbaceous Plant Hydrolysates Using Metabolically Engineered *Corynebacterium glutamicum*^§^

**DOI:** 10.17113/ftb.63.02.25.8808

**Published:** 2025-06

**Authors:** Dae-Seok Lee, Eun Jin Cho, Seryung Kim, Dien Thanh Nguyen, Hyeun-Jong Bae

**Affiliations:** 1 Bio-Energy Research Institute, Chonnam National University, Gwangju 61186, Republic of Korea; 2 Department of Integrative Food, Bioscience and Biotechnology, Chonnam National University, Gwangju 61186, Republic of Korea; 3 School of Biotechnology, Tan Tao University, Long An 82000, Viet Nam

**Keywords:** succinic acid, lignocellulosic biomass, bamboo, enzymatic saccharification, fermentation

## Abstract

**Research background:**

Succinic acid from lignocellulosic biomass is a sustainable alternative for biochemical production that is an environmentally friendly substitute for petroleum-based chemicals. The aim of this study is to evaluate the effects of variations in hemicellulose content and cellulose fibre structure within the microfibrils of woody and herbaceous plants on the enzymatic saccharification and succinic acid production efficiency of Psod:*SucE*12-*ΔldhA*, a strain overexpressing the succinic acid transporter (*SucE*).

**Experimental approach:**

The study investigated the influence of different monosaccharide combinations on succinic acid production, focusing on combinations with mannose compared to glucose alone. Additionally, hydrolysates from different lignocellulosic biomass — bamboo, oak, poplar, pine and spent coffee grounds — were analysed to determine the most favourable bioresource for succinic acid production.

**Results and conclusions:**

Monosaccharide combinations containing mannose resulted in 2.20-2.48 times higher succinic acid production than glucose alone, indicating a positive influence of mannose on succinic acid metabolism. Among the lignocellulosic biomass hydrolysates, bamboo, with its higher xylose content than woody plants, was the most efficient bioresource for succinic acid production (23.38–24.12 g/L within 24 h), followed by oak, poplar, pine and spent coffee grounds. Therefore, improving the xylose consumption rate is crucial for increasing succinic acid production from lignocellulosic biomass and increasing market competitiveness.

**Novelty and scientific contribution:**

This research emphasises the potential of lignocellulosic biomass, especially bamboo, as a sustainable feedstock for succinic acid production. The novelty of the study lies in the detailed investigation of how hemicellulose content and cellulose fibre structure affect enzymatic saccharification and fermentation. The significant influence of mannose and xylose on the succinic acid yield provides key insights for the optimisation of biomass use in biochemical production. These findings promote bio-based chemical production, reduce reliance on fossil fuels and improve industrial sustainability.

## INTRODUCTION

Amid global concern about the environmental impact of CO_2_ emissions and microplastics associated with a petroleum-based economy, biochemicals have attracted considerable attention as a potential remedy. In particular, succinic acid, which has diverse applications in various industrial fields, stands out as an important platform chemical for the bio-based economy.

Succinic acid, a member of the four-carbon dicarboxylic acid family, serves as an intermediate in the citric acid and glyoxylate cycle during the conversion of glucose to adenosine triphosphate (ATP) and nicotinamide adenine dinucleotide phosphate (NADPH) in living cells. Due to its well-defined structural properties, it has many applications in pharmacy, agriculture, cosmetics and food industry. Notably, succinic acid can be polymerised to produce diverse industrial products such as polyurethanes. It can also be converted to 1,4-butanediol (1,4-BDO), which serves as a precursor for the synthesis of polyesters, polybutylene succinate (PBS) and tetrahydrofuran (THF). After the COVID-19 pandemic, bioactive compounds such as vitamins and antioxidants have gained attention for supporting the immune system and overall health. Succinic acid, with its antioxidant properties, has emerged as a promising bioactive compound in the food industry (*1*, *2*).

Succinic acid has been reported to be produced by wild-type microbes (*e.g*. *Actinobacillus succinogenes*) and recombinant strains of various microorganisms, including *Escherichia coli*, *Corynebacterium glutamicum*, *Mannheimia succiniciproducens* and *Yarrowia lipolytica* (*3*-*7*). Bio-based succinic acid production offers several advantages, including the availability of various fermentable sugar resources, high fermentation efficiency and the usability of intermediates and products (*6*). However, challenges remain in terms of cost competitiveness on the market compared to petroleum-based succinic acid production. A minimum productivity of 2.5 g/(L∙h) succinic acid is required, especially when using corn starch as a raw material (*8*). Above all, the estimation of greenhouse gas emissions during succinic acid production from lignocellulosic biomass, which is currently limited to a laboratory scale, has not yet been reported. While corn starch-based succinic acid production has its merits compared to lignocellulosic biomass-based production, there are some problems associated with the use of corn starch as an alternative carbon source for biochemicals or bioenergy. One issue is the rising price of corn stock due to its excessive utilization, which leads to competition with animal feedstock and food chains on the market. Other challenges include the extensive need for land, fertilizers and pesticides for farming corn crops. These reasons explain why the companies that produce succinic acid from corn-based carbon sources are predominantly located in China and the USA. The drawbacks of corn starch-based succinic acid shift the interest of alternative bioresources to lignocellulosic biomass, which is an abundant, carbon-neutral and renewable source worldwide (*9*).

*C. glutamicum* is a Gram-positive soil bacterium, well known as an industrial microbe with a wide range of applications, including the production of amino acids, organic acids, fuels and healthcare products (*10*). Briki *et al*. (*11*) reported that wild-type *C. glutamicum* is a natural overproducer of succinic acid under the optimal transition conditions with a volumetric mass transfer coefficient (*k*_L_a value) of 5 h^-1^. Numerous research that uses metabolic engineering through knock-out of genes involving the metabolism of lactic and acetic acids, and overexpression of genes associated with citric acid and glyoxylate cycles has been reported (*3*, *12*, *13*). Moreover, the presence of the gene (*SucE*) encoding the transporter of succinic acid in *C. glutamicum*, characterised as one-way exporter from the cell into the medium, provides a significant advantage to *C. glutamicum* during succinic acid fermentation (*14*).

Metabolic engineering aimed to increase succinic acid production by *C. glutamicum* has been conducted using allelic exchange, transposon and integrase-mediated integration. However, these methods are known for low efficiency of homologous recombination and the potential for inaccurate genome editing (*15*). Clustered regularly interspaced short palindromic repeats (CRISPR) gene editing system has been proposed as a solution to tackle these challenges. CRISPR-Cpf1 (Clustered regularly interspaced short palindromic repeats and CRISPR nuclease from *Prevotella* and *Francisella 1*) was identified as a type V CRISPR with a nuclease consisting of 1 200 500 amino acids (*16*). The enzyme recognises a protospacer adjacent motif (PAM) site of 5’ (T)TTN 3’, flanking 24 base pairs of the target genomic DNA, and guides the annealing of crRNA (CRISPR RNA) with the complementary target DNA. The double-stranded DNA cleavage can induce the activation of endogenous DNA repair systems, namely non-homologous end-joining (NHEJ) and homologous recombination (HR). The combination of CRISPR-Cpf1 with HR DNA repair system can enable more precise nucleotide or gene substitution, insertion and deletion (*17*). CRISPR-Cpf1 genome editing system was successfully applied for nucleotide substitutions in the gene encoding arginine repressor (argR), as well as simultaneous insertions and deletion in the gene of C50 carotenoid epsilon cyclase (crtYf) of *C. glutamicum*.

Most of the previous research using genetically engineered *C. glutamicum* for succinic acid production utilized pure glucose as the sole carbon source (*17*), except for a few cases where corn cob hydrolysate was used (*13*). Lignocellulosic biomass is a good carbon source for succinic acid production. Depending on the types of wood and herbaceous plants, lignocellulosic biomass consists of various monosaccharides such as glucose, xylose, mannose, galactose, arabinose, *etc*. (*18*-*20*). The impact of these monosaccharides and hydrolysates from lignocellulosic biomass on the production of succinic acid and other metabolites during the fermentation of *C. glutamicum* remains largely unexplored. In this study, the metabolically engineered *C. glutamicum* strains created through CRISPR/Cpf1 gene editing system were applied to produce succinic acid from monosaccharides (glucose, xylose and mannose), hydrolysates of three woody plants (pine, oak and poplar) and two herbaceous plants (bamboo and spent coffee grounds). It elucidates the effect of monosaccharides on succinic acid fermentation and determines the optimal biomass and hydrolysate concentration for efficient succinic acid production.

## MATERIALS AND METHODS

### Preparation of wood materials and chemicals

Pine, poplar oak and bamboo were obtained from the arboretum at Chonnam National University, Gwangju, South Korea. Pine (*Pinus densiflora*, *d*=35 cm), poplar (*Populus deltoids*, *d*=20 cm) and oak (*Quercus acutissima*, *d*=25 cm) wood were cut into chips of approx. 0.25 cm×0.35 cm×5.0 cm (width, height and length, respectively) using a saw. Bamboo shoots (*Phyllostachs pubescens*) were cut into 5 cm columns and then vertically divided into eight segments (*21*). Spent coffee grounds were sourced from Starbucks in Gwangju, South Korea.

All chemicals were purchased from Sigma-Aldrich, Merck (St. Louis, MO, USA).

### Pretreatment and enzymatic hydrolysis

The wood samples were individually soaked in the hydrogen peroxide-acetic acid (HPAC) solution containing *φ*(30 % H_2_O_2_,HAc)=50 % and then incubated at 80 °C for 3−4 h (*17*). The wood fibres were filtered using an iron mesh tray and then washed with water several times until the lignin was completely removed using HPAC solution. Subsequently, spent coffee grounds were individually treated with 1 M NaOH, 6 % H_2_O_2_ and a combination of both at room temperature for 24 h. After washing with water, the HPAC-pretreated woody and herbaceous samples were freeze-dried and stored at room temperature.

The HPAC-pretreated samples (1−4, 5, 10 and 20 %) were hydrolyzed with 20 filter paper units (FPU) per g biomass cellulase (Cellic CTec2; Novozymes, Bagsvaerd, Denmark) at 50 °C for 5 days. The resulting hydrolysates were centrifuged (Avanti J-E; Beckman Coulter Inc, CA, USA) at 25 600×*g* (15 000 rpm) to separate fibre debris and soluble sugars and then filtered using Whatman No. 1 paper. The concentration of reducing sugars in each hydrolysate was measured using the DNS assay (consisting of 10 g/L dinitrosalicylic acid, 2 % NaOH, 0.5 g/L sodium sulfite, Rochelle salt (KNaC_4_H_4_O_6_·H_2_O) and 2 mL/L phenol). The hydrolysis ratio was calculated using the following equation:


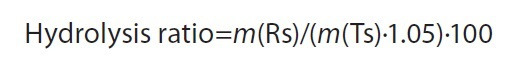
 /1/

where Rs is the reducing sugar (g) in the hydrolysate, Ts is the total monosaccharide (g) in biomass and 1.05 is the mass conversion factor that accounts for the increase in mass when polysaccharides are hydrolyzed into monosaccharides.

### Preparation of Corynebacterium glutamicum strain for transformation

The strain of *C. glutamicum* (ATCC 13032) was obtained from the Korean Agricultural Culture Collection (KACC). The cells were cultured in Luria-Bertani (LB) liquid medium (BioShop, Burlington, Canada) containing 20 g/L LB broth at 30 °C for 24~48 h for the preparation of competent cells. The microbes were harvested and re-cultured in 100 mL nucleic cell medium (NCM) containing 1.0 g/L yeast extract, 5 g/L tryptone, 5 g/L glucose, 17.4 g/L K_2_HPO_4_, 0.05 g/L MgSO_4_·7H_2_O, 0.3 g/L trisodium citrate, 91.1 g/L sorbitol and 11.6 g/L NaCl, pH=7.2) at 30 °C for 4 h. After centrifugation at 140×*g* (3500 rpm) using an Avanti J-E centrifuge (Beckman Coulter Inc, CA, USA), the cells were rinsed three times with ice-cold 10 % glycerol and resuspended also with ice-cold 10 % glycerol. The cells (90 μL) were aliquoted into microcentrifuge tubes and stored at - 80 °C.

Plasmid DNA (5–10 μL) was added to a tube containing 90 μL of competent cell solution and transferred to a 2-mm electroporation cuvette (Sigma-Aldrich, Merck). Electroporation was performed with a MicroPulser system (BioRad, Hercules, CA, USA) at 1.8 kV for 5 ms. A volume of 900 μL of brain heart infusion supplemented (BHIS) liquid solution, containing 18 g/L brain heart infusion (BHI) and 91 g/L sorbitol, was added and transferred to microcentrifuge tubes. The cells were immediately incubated at 46 °C for 6–15 min. The cells were then plated on LB medium supplemented with LBHIS (2.5 g/L yeast extract, 5 g/L tryptone, 5 g/L NaCl, 18.5 g/L BHI, 91 g/L sorbitol, 18 g/L agar, pH=7.2) containing 50 μg/mL kanamycin or 30 μg/mL streptomycin and incubated at 30 °C until colonies appeared.

### CRISPR-Cpf1-mediated recombination of Corynebacterium glutamicum

**Fig. 1:**
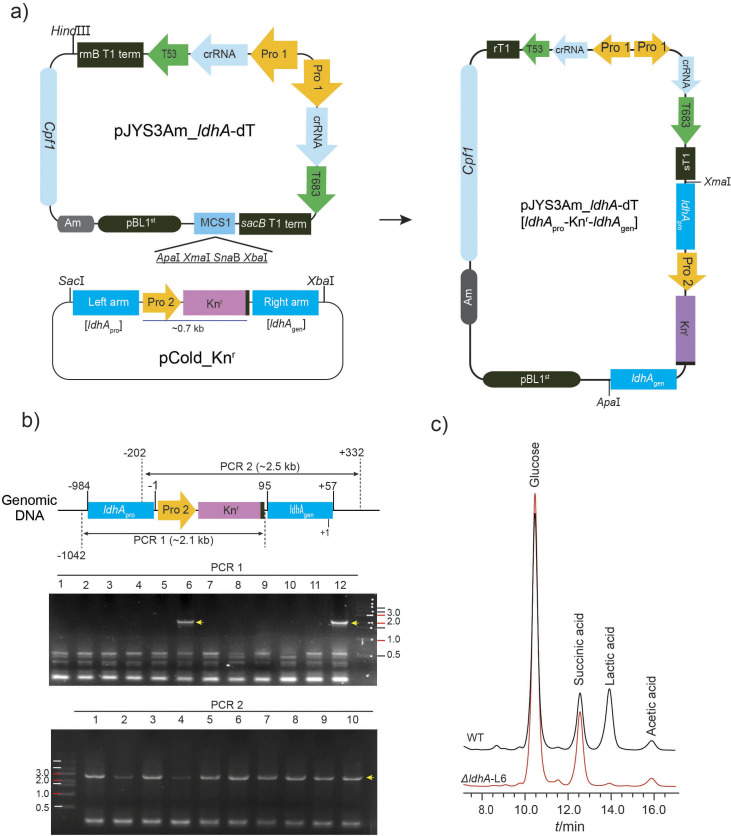
Construction of a homologous recombination vector for CRISPR/Cpf1 genome editing: a) the double target DNA sets (T53 and T683) with crRNAs were introduced into the pJYS3Am vector. The homologous arms consisted of the promoter region of the *ldhA* gene (left arm) and the open reading frame of *ldhA* (right arm). A spontaneous deletion and overexpression vector, pJYS3Am_*ldhA*-dT [*ldhA*_pro_-Kn^r^-*ldhA*_gen_], was constructed, b) when the 1^st^ and 2^nd^ homologous crossovers were complete, a 95-bp-long region of the *ldhA* gene was deleted. PCR 1 and 2 show the complete homologous recombination, and c) the *ΔldhA-L6* strain was used for fermentation with pure glucose and the activity of *ldhA* was completely blocked. Kn^r^=kanamycin resistance gene, Am^r^=ampicillin resistance gene, rmB T1 term and sacB T1 term=terminator regions of the rmB and sacB genes, respectively; Pro 1=J23119 promoter, Pro 2=Kn^r^ promoter, MCS=multiple cloning sites, WT=wild type

**Fig. S1:**
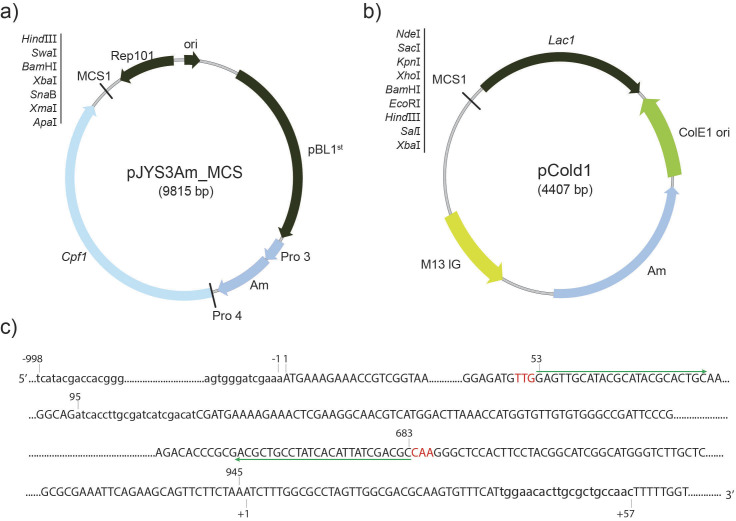
The vectors used for generating CRISPR/Cpf1 system and for homologous recombination: a) the pJYS3_Am_MCS vector was derived from the pJYS3_ΔcrtYF plasmid, which was obtained from Addgene (www.addgene.org), b) the pCold vector (TaKaRa) was used as an intermediate vector to construct homologous arms and the expression gene set for the pJYS3Am_*ldhA*-dT [*ldhA*_pro_-Kn^r^-*ldhA*_gen_] and pJYS3Am_Kn^r^-sT [*ldhA*_pro_-Psod:*SucE*-rspLm-*ldhA*_gen_] vectors, and c) the lactate dehydrogenase A (*ldhA*) gene and its flanking regions were used as homologous arms and two target DNA sequences for CRISPR/Cpf1 genome editing in *Corynebacterium glutamicum*. Lowercase letters indicate the primer annealing sites for PCR amplification of the promoter region (-1 to -998) and the region of the *ldhA* gene (95 to 945) along with a partial terminator region (+1 to +57). Capital letters denote the *ldhA* gene. Red letters represent PAM sites and green arrows indicate the target DNA sequences

The CRISPR-Cpf1 vector (pJYS3_∆crtYF), obtained from Addgene Co. (Watertown, MA, USA), was modified by replacing the kanamycin resistance gene with an ampicillin resistance marker gene and adding a multiple cloning site with restriction enzymes. The modified vector pJYS3Am_MCS is shown in Fig. 1. Information regarding the primers used in this study is given in Table S1. The promoter and crRNA linked to the 24-bp long target DNA sequence (T53 and T683) on the lactate dehydrogenase A (*ldhA*) gene were sequentially and individually incorporated to generate pJYS3Am_*ldhA*-dT. pCold vector was used to construct the homologous recombination cassette, [left arm-Pro4:Kn^r^-Right arm] (Fig. 1 and Fig. S1). Finally, the homologous recombination cassette was introduced into the pJYS3Am_*ldhA*-dT vector at the sites of Xma1−Apa1, generating pJYS3Am_*ldhA*-dT-[*ldhA*_pro_-Pro2:Kn^r^-*ldhA*_gen_]. The CRISPR-Cpf1 vector was transformed into the wild-type competent cells of *C. glutamicum* using an electroporation machine (MicroPulser, BioRad) set at 1.8 kV for 5 ms (*22*). Subsequently, the knock-out mutant of *ldhA* (*ΔldhA-L6*) was selected, and the first and second homologous recombination events were confirmed by polymerase chain reaction (PCR) analysis (described in *Genomic DNA isolation and PCR analysis*).

**Fig. 2:**
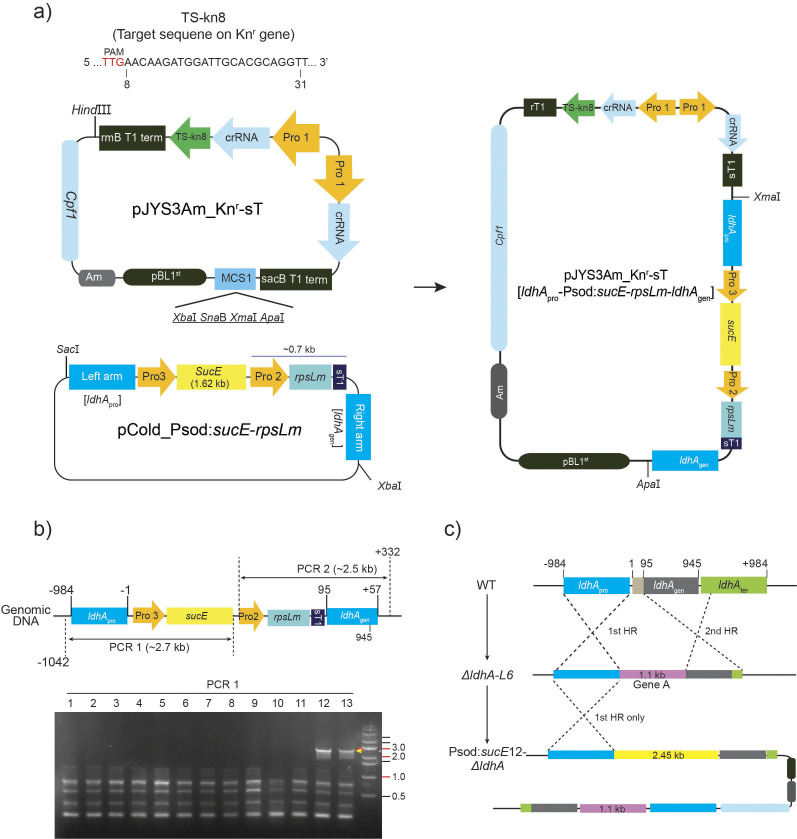
Overexpression of succinic acid transporter (*SucE*) using a CRISPR/Cpf1 vector containing a single-target DNA set: a) the target site was located at 8 bp from the start codon of the Kn^r^ gene in the *ΔldhA-L6* strain. The *SucE* and *rpsLm* genes between the left and right arms were subcloned to construct the CRISPR/Cpf1 single target vector pJYS3Am_Kn^r^_sT [*ldhA*_pro_-Psod:*SucE*-rpsLm-*ldhA*_gen_]. The expression of the rpsLm gene is regulated by the Kn^r^ promoter, providing streptomycin resistance to the *ΔldhA-L6* strain, b and c) the overexpression strain Psod:*SucE*12-*ΔldhA* was created and the occurrence of the 1^st^ homologous crossover was confirmed. However, the 2^nd^ crossover was not observed. Pro3=Psod promoter, *rpsLm*=ribosomal S12 protein mutant gene, rT1 and sT1=rmB T1 and sacB T1 terminators, respectively; HR=homologous recombination

The succinic acid transporter-overexpressing strain, Psod:*SucE-ΔldhA*, was generated by transforming the single-target CRISPR vector, pJYS3Am_Kn-sT, containing the homologous recombination set [*LdhA*_pro_-Psod:*SucE*-rspLm-*LdhA*_gen_], into the *ΔldhA-L6* knock-out strain (Fig. 2). The succinic acid transporter (*SucE*) and ribosomal S12 protein gene (*rpsL*) were isolated from wild-type *C. glutamicum* (described in the section *Genomic DNA isolation and PCR analysis*). The AAG (Lys^43^) sequence on the *rpsLm* gene was replaced with AGG (Glu), thereby generating the streptomycin resistance gene, *rpsLm*. The *SucE* and *rspLm* genes were subcloned downstream of the Psod and Kn^r^ promoters on the pCold vector, respectively, resulting in the pCold homologous recombination set: pCold [*LdhA*_pro_-Psod:*SucE*-rspLm-*LdhA*_gen_]. The 4.95 kb-long homologous recombination set was amplified with PCR and introduced into the pJYS3Am_Kn-sT vector to generate pJYS3Am_Kn-sT [*LdhA*_pro_-Psod:*SucE*-rspLm-*LdhA*_gen_]. Transformation was performed following the previously described procedure and two transformants (Psod:*SucE-ΔldhA*) were selected on solid LB medium supplemented with 30 μg/mL streptomycin.

### Genomic DNA isolation and PCR analysis

The colonies of *C. glutamicum* obtained on LB or selection media were inoculated into 5 mL of liquid LB medium and then incubated at 30 °C overnight. The cells were then harvested, suspended in 100 μL extraction buffer (0.2 M lithium acetate, 1 % sodium dodecyl sulfate (SDS)) and incubated at 70 °C for 10 min. The genomic DNA was then precipitated by adding 300 μL ethanol and vortexing the samples. Next, cell debris and genomic DNA were separated from the liquid phase by centrifugation (Avanti J-E; Beckman Coulter Inc) at

140×*g* (3500 rpm) for 10 min. The precipitate was dried at room temperature and then dissolved in 100 μL of distilled water. The cell debris was then removed by centrifuging at 140×*g* (3500 rpm) for 10 min, and 1 μL of the supernatant was used to isolate the left and right arms of the *LdhA*, *SucE* and *rspL* genes or to verify the selection of the transformants using PCR analysis and the primers listed in Table S1.

### Succinic acid production from the hydrolysates

The colonies of the *ΔldhA-L6* knock-out strain or the Psod:*SucE-ΔldhA* overexpression strain line 12 (Psod:*SucE*12-*ΔldhA*), obtained on the selectied media, were separately inoculated into 20 mL of liquid LB medium and incubated at 30 °C for 24 h. After that, the cells were transferred into 1 L of liquid LB medium and incubated at 30 °C for 2 days. The harvested cells (approx. 10 g/L cell dry mass) were used to produce succinic acid from the hydrolysates of pine, poplar, oak, bamboo and spent coffee grounds. Mineral salts (0.5 g/L K_2_HPO_4_, 0.5 g/L KH_2_PO_4_, 0.5 g/L MgSO_4_·7H_2_O, 4.2 mg/L MnSO_4_·7H_2_O and 6.0 mg/L FeSO_4_·7H_2_O) and vitamins 0.2 mg/L biotin and 0.2 mg/L thiamine were added to each of the hydrolysates and the pH was adjusted to 7.5. The cells were suspended in 100 mL of each hydrolysate, taken in a cylindrical bottle (volume 700 mL) and finally, 0.4 M NaHCO_3_ was added to each suspension. Each cylindrical bottle was sealed with a screw cap and fermentation was carried out at 200 rpm and 30 °C in a shaking incubator (VS-8480; Vision Science, Daegu, South Korea). Samples were taken after 3, 6, 9 and 24 h. Volumetric mass transfer coefficient (*k*_L_a), which can provide information about the operation parameters during the experiment, was calculated using the following equation (*11*):


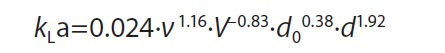
 /2/

where 0.024 is an empirical constant, *ν* is the shaking frequency (rpm), *V* is the filling volume (%), *d* is the maximal shake flask diameter (cm) and *d*_0_ is the shaking diameter. The *k*_L_a value for this experiment was determined to be 9.6 h^-1^. The concentrations of metabolites and monosaccharides were analysed using high-performance liquid chromatography (HPLC) with a 2702 Autosampler (Waters, Milford, MA, USA).

To compare the succinic acid production efficiency based on reducing sugar concentrations, the reducing sugar sets (RS20, RS30, RS40 and RS50 corresponding to the hydrolysate concentrations of 20, 30, 40 and 50 g/L, respectively) were prepared by dilution of hydrolysates from pine, oak, poplar, bamboo and spent coffee grounds, respectively. Similarly, succinic acid was also produced using the strain Psod:*SucE*12-*ΔldhA* (*γ*(cell dry mass)=10 g/L).

### Fermentation of the monosaccharides using the Corynebacterium glutamicum strains

The *ΔldhA-L6* and Psod:*SucE*12-*ΔldhA* strains (*γ*(cell dry mass)=10 g/L) were individually used to ferment 10 and 20 g/L glucose (G10 and G20), 20 g/L xylose (X20) and 20 g/L mannose (M20) in separate 50-mL conical tubes (*d*=3 cm) containing 10 mL solution of mineral salts. Similarly, the following combinations of monosaccharides were also fermented: 20 g/L glucose with 10 g/L xylose (G20X10), 20 g/L glucose with 10 g/L mannose (G20M10), 20 g/L glucose with 5 g/L xylose and 5 g/L mannose (G20X5M5), 20 g/L glucose with 5 g/L xylose and 10 g/L mannose (G20X5M10), 20 g/L glucose with 10 g/L xylose and 5 g/L mannose (G20X10M5), and 20 g/L xylose with 10 g/L mannose (X20M10). All conical tubes were sealed after inoculation with the respective strain and fermented at 200 rpm and 30 °C in a shaking incubator (VS-8480; Vision Science). Samples were taken after 3, 6, 9 and 24 h. The concentrations of metabolites and monosaccharides were measured by HPLC analysis. The *k*_L_a was calculated using Eq 2. For this experiment, the *k*_L_a value was 16.3 h^-1^. The yield and conversion efficiency of succinic acid were calculated using the following equations:


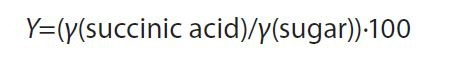
 /3/


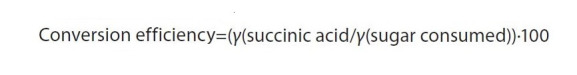
 /4/

The amount of succinic acid measured 24 h after the fermentation was used for the calculation.

### Measurement of metabolites and monosaccharides

The concentrations of organic acids, glucose and xylose were measured using an HPLC system (2702 Autosampler; Waters) equipped with a refractive index (RI) detector (Waters 2414). A reversed-phase octadecylsilane (ROA) column (7.8 mm×300 mm; Phenomenex, Torrance, CA, USA) was used to determine the amount of organic acids, glucose, xylose and mannose in the fermented solution, while an RPM column (4.6 mm×300 mm; Phenomenex) was used to analyse the amount of monosaccharides in the hydrolysates. The mobile phase, consisting of 5 mM sulfuric acid, was passed through the column at a flow rate of 0.6 mL per min. The temperatures of the column and detector were set at 65 and 40 °C, respectively.

### Analysis of the chemical composition of lignocellulosic biomass

The monosaccharides, including glucose, xylose and mannose, in the HPAC-pretreated biomass (pine, poplar, oak, bamboo and spent coffee grounds) were quantified using gas chromatography, following a previously established method (*21*). Each raw and HPAC-pretreated biomass were treated with 0.25 mL of *φ*(H_2_SO_4_)=72 % for 45 min at 30 °C and diluted with distilled water to *φ*(H_2_SO_4_)=4 %. The hydrolysis step was carried out at 121 °C for 1 h and a solution containing a known amount of myo-inositol was used as an internal standard. The solution was then neutralised with ammonia water. A volume of 1 mL of 2 % (*m*/*V*) sodium borohydride dissolved in dimethyl sulfoxide and 0.1 mL glacial (anhydrous) acetic acid (18 M) were added to degrade the sodium tetrahydroborate. Then, 0.2 mL of methyl imidazole and 2 mL of anhydrous acetic acid were added sequentially followed by 5 mL of deionized water and 2 mL of dichloromethane for extraction. The samples were analysed using gas chromatography (GC-2010; Shimadzu, Otsu, Japan) and a DB-225 capillary column (30 m×0.25 mm i.d., 0.25 μm film thickness, J&W; Agilent Technologies, Folsom, CA, USA) operated with helium. The operating conditions were as follows: injector temperature of 220 °C, flame ionization detector (FID) at 250 °C, and an oven temperature of 110 °C for 1.5 min with a constant increase of 10 °C/min to 220 °C.

## RESULTS AND DISCUSSION

### Generation of Corynebacterium glutamicum recombinants

**Fig. S2:**
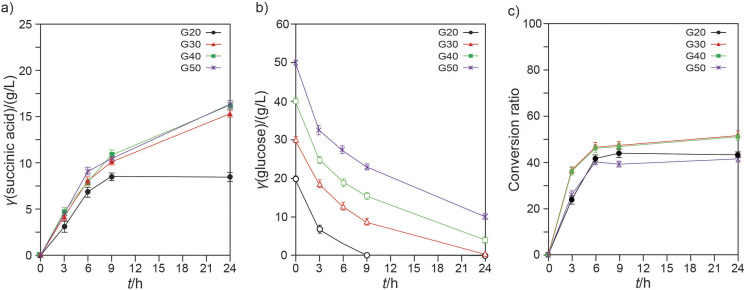
Succinic acid production depends on glucose concentration: a) succinic acid production, b) glucose consumption, and c) the conversion ratio. G20, 30, 40 and 50=20, 30, 40 and 50 g/L of glucose

Under oxygen-deprived or anaerobic conditions, wild-type *Corynebacterium glutamicum* primarily excretes lactic acid, which is approx. 2.5 times more abundant than succinic acid during the fermentation of pure glucose at an optimal *k*_L_a of 9.6 h^-1^ for 50 h (*11*). Therefore, the gene encoding lactate dehydrogenase A (*ldhA*) was first targeted and blocked using the CRISPR/Cpf1 genome editing system to increase succinic acid production (Fig. S2). The two target DNAs on the *ldhA* gene and the homologous recombination cassette (*LdhA*_pro_-Kn^r^-*LdhA*_gen_) were inserted into the pJYS3Am_*ldhA*-dT vector, constructing the all-in-one vector, pJYS3Am_*ldhA*-dT-[*LdhA*_pro_-Kn^r^-*LdhA*_gen_] (Fig. 1 and Fig. S1). This vector was transformed into the wild-type *C. glutamicum* to create an *ldhA* gene knock-out mutant *ΔldhA-L6* strain (Fig. 1). After confirming the PCR1 fragment for the first homologous recombination, the colony no. 6 (from PCR 1) was incubated at 46 °C to induce the second homologous recombination. It was confirmed that the deletion in the genomic DNA corresponded to a region approx. 94 bp from the start codon of the *ldhA* gene and the expression of the *ldhA* gene was successfully blocked through a frame shift. During the fermentation of pure glucose, the *ΔldhA-L6* strain excreted succinic acid as the main organic acid due to the absence of lactic acid in the medium.

The succinic acid transporter of *C. glutamicum*, *SucE*, is a unidirectional transport system responsible for exporting succinic acid to the external environment (*14*). It offers a significant advantage in boosting the production of succinic acid through gene overexpression compared to other succinic acid transporter systems, such as the Dcu family in *E. coli*. Notably, extracellular succinic acid has been reported to inhibit both glucose consumption rate and succinic acid production (*3*). Considering the potential inhibitory effect of intracellular succinic acid on the metabolic pathway of succinic acid, including glycolysis, the glyoxylate cycle and the citric acid cycle, it can be hypothesised that a stronger excretion of succinic acid may lead to an increase in the glucose-to-succinic acid conversion rate. To investigate this hypothesis, the single-target DNA sequence of the kanamycin resistance gene located in the genomic DNA of the *ΔldhA-L6* strain and the homologous recombination set were subcloned to generate the CRISPR/Cpf1 genome editing vector, pJYS3Am_Kn^r^-sT [*LdhA*_pro_-Psod:*SucE*-rsplm-*LdhA*_gen_] (Fig. 2). Consequently, a *SucE* overexpressing strain regulated by the constitutive Psod promoter, namely the Psod:*SucE*E12-*ΔldhA* strain was generated.

Allelic exchange through homologous recombination has been effectively utilised in *C. glutamicum* for metabolic engineering purposes (*15*). The successful deletion of a gene from the genomic DNA involves sequential first and second crossovers in the process of homologous recombination. Notably, the spontaneous process of gene deletion and insertion is more successful when the size of the insertion gene is approx. 1.0 kb or below. Thus, spontaneous gene deletion (95 bp of *LdhA* gene) and insertion (1.1 kb of kanamycin resistance gene) were observed in the *ΔldhA-L6* mutant under serial heat shock and crossovers. However, in the Psod:*SucE*E12-*ΔldhA* transformant, colonies showing complete crossovers could not be selected. It is presumed that Psod:*SucE*12-*ΔldhA* transformant, containing a 2.3-kb insertion, predominantly undergoes the first crossover or infrequently achieves the second crossover recombination, posing a significant challenge in the selection of colonies with complete crossovers.

### Saccharification of lignocellulosic biomass

Lignocellulosic biomass encompasses a diverse array of carbohydrates and their composition can vary based on the species, even within the same plant. This variability can be attributed to variations in growing conditions, growth stage and tissue position within the plant (*23*). Cellulose, a major component, consists of glucose, while hemicellulose is a heterogeneous mixture of various sugars, including xylose, mannose, galactose, arabinose and others. In softwood, hemicellulose contains arabinoglucuronoxylan and galactoglucomannan, responsible for 8 and 18 % of the cell wall components, respectively, while in hardwood, hemicellulose consists of glucuronoxylan and glucomannan, which account for 15−30 % and 2−5 % of the cell wall components, respectively (*24*). The hemicellulose in Korean red pine (*Pinus densiflora*) contains 14.6 % galactomannan and 6.4 % xylan (*25*). The cell wall of herbaceous plants contains a primary cell wall rich in pectin, which contributes to the release of more diverse sugars in the hydrolysate (*26*). The diversity of cell wall components in lignocellulosic biomass facilitates the determination of sugar preferences of *C. glutamicum* during succinic acid fermentation. In this study, the *ΔldhA* strain served as a standard and represented the baseline response to various sugars derived from lignocellulosic biomass. To represent different plant species, we selected five biomass samples: pine wood for softwood, poplar and oak wood for hardwood and bamboo and spent coffee grounds for herbaceous plants. The mass fraction of carbohydrate in the biomass is shown in Table 1 (*23*, *24*, *27*-*31*). Xylose is the main component of hemicellulose in poplar, oak and bamboo. In pine and spent coffee grounds, mannose is the main component of hemicellulose, accounting for 17.53 and 44.57 % of the carbohydrates, respectively. Additionally, both pine and spent coffee grounds contain higher mass fractions of galactose than the other biomass samples. The HPAC-pretreated pine, poplar, oak, bamboo and spent coffee grounds were enzymatically hydrolyzed with a cellulase cocktail solution (20 FPU per g biomass) at 50 °C for 5 days, resulting in hydrolysis ratios from 80 to 98 %, except spent coffee grounds (Table 2).

**Table 1:** The chemical composition of lignocellulosic biomass

	*w*(cell wall)/%			Carbohydrate						
Lignocellulosic biomass	Cellulose	Hemi- Cellulose	Lignin	Glu *w*/(g/kg), (w/%)	Xyl *w*/(g/kg), (*w*/%)	Ara *w*/(g/kg), (*w*/%)	Rham *w*/(g/kg), (*w*/%)	Man *w*/(g/kg), (*w*/%)	Gal *w*/(g/kg), (*w*/%)	Total *w*/(g/kg), (*w*/%)
Softwood (*27*)	45−50	25−35	18−25	(67.06)	(9.49)	(2.41)	-	(17.18)	(3.86)	(100)
Pine (*28*)	42.10	21.80	24.12	(65.88)	(9.23)	(2.66)	-	(17.53)	(4.69)	(100)
HPAC-pretreated pine	51.2	8.3	-	489± 46 (86.0±0.2)	11.0±1.4 (1.93±0.09)	0.5±0.1 (0.10±0.02)	- -	66.3±5.8 (11.7±0.1)	1.8±0.2 (0.31±0.05)	569±53 (100.00)
Hardwood (*24*, *29*)	44−55	24−40	18−25	(67.82)	(26.19)	(0.80)	-	(3.59)	(1.60)	(100)
Poplar (*28*)	42.90	20.25	23.60	(67.93)	(25.73)	(0.55)	-	(2.65)	(0.80)	(100)
HPAC-pretreated poplar	54.4	5.9	-	544±44 (90.3±0.6)	38.7±6.1 (6.4±0.5)	1.2±0.4 (0.20±0.05)	-	18.64±2.0 (3.1± 0.1)	- -	602±52 (100.00)
Oak (*24*)	39.71	28.77	23.99	(64.47)	(29.87)	(0.63)	-	(3.14)	(1.89)	(100)
HPAC-pretreated oak	52.4	4.4	-	524±62 (92.2± 0.1)	34.5±4.7 (6.1± 0.1)	1.0±0.2 (0.18±0.02)	- -	5.8±0.3 (1.03±0.07)	3.1±0.6 (0.5±0.1)	568±68 (100.00)
Herbaceous plants (*30*)	28~40	19~35	7−32	-	-	-	-	-	-	-
Bamboo (*23*)	50.81	38.73	10.47	428 ±18 (56.84)	300.2± 9.8 (39.84)	15.5±0.7 (2.05)	2.17±0.39 (0.29)	4.4±2.8 (0.58)	3.7± 1.0 (0.49)	754±18 (100.00)
HPAC-pretreated Bamboo	67.77	7.06	-	678± 62 (90.5± 0.4)	64.1± 4.2 (8.6± 0.3)	2.4±0.3 (0.32±0.06)	-	4.1±0.3 (0.55±0.05)	-	748± 66 (100.00)
Spent coffee grounds (*31*)	9.90	33.40	38.60	(22.86)	(2.54)	(5.08)	-	(44.57)	(24.94)	(100)
Pretreated-spent coffee grounds	17.58	32.28	-	175.5±9.2 (35.2±0.4)	0.00 (0.00)	0.94±0.04 (0.19±0.00)	-	308±12. (61.9±0.2)	13.5±0.2 (2.7±0.2)	498.3±1.5 (100.00)

The numbers in parentheses for carbohydrates indicate their ratios, which have been partially adjusted to enable a consistent comparison of the sugar components among the biomass results. HPAC=hydrogen peroxide-acetic acid solution, Glu=glucose, Xyl=xylose, Ara=arabinose, Rham=rhamnose, Man=mannose, Gal=galactose

**Table 2:** Enzymatic hydrolysis ratios of different lignocellulosic biomass

	Hydrolysis ratio/%				
*w*(lignocellulosic biomass)/%	Pine	Poplar	Oak	Bamboo	Spent coffee grounds
1	92.1±1.2	86.6±1.2	78.3±0.1	93.2±1.3	79.1±1.8
2	94.0±2.0	93.2±1.3	84.7±2.5	96.2±1.9	78.7±3.9
3	90.5±1.6	96.0±1.3	80.9±1.5	98.5±3.4	66.7±2.6
4	83.7±3.3	94.3±5.0	87.7±3.2	91.5±1.6	59.7±4.5
5	79.2±3.0	90.6±2.1	87.1±2.2	90.7±4.2	53.9±7.6

### Succinic acid production from lignocellulosic biomass using the knock-out strain ΔldhA

**Fig. 3:**
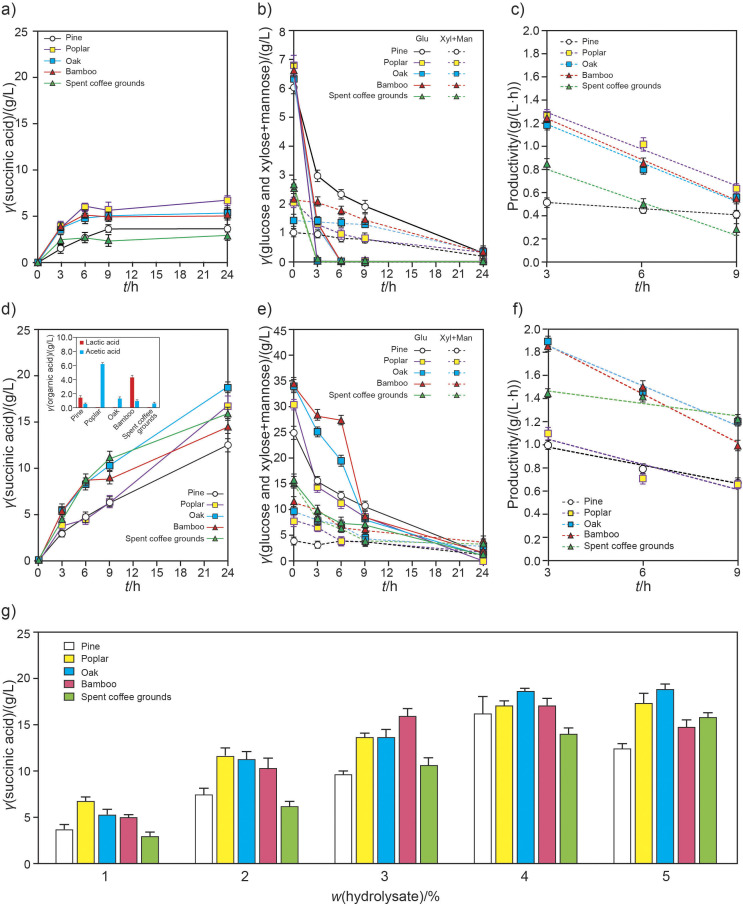
Succinic acid production from woody plants (pine, poplar and oak) and herbaceous plants (bamboo and spent coffee grounds) using *ΔldhA-L6* strain: a) succinic acid production using 1 % hydrolysate under unsaturated sugar conditions, b) monosaccharide consumption of 1 % hydrolysate under unsaturated sugar conditions, c) productivity of 1 % hydrolysate under unsaturated sugar conditions, d) succinic acid production using 5 % hydrolysate under saturated sugar conditions, e) monosaccharide consumption of 5 % hydrolysate under saturated sugar conditions, f) productivity of 5 % hydrolysate under saturated sugar conditions, and g) a comparison of succinic acid production patterns depending on different sugar concentrations and compositions derived from lignocellulose biomass after 24 h of fermentation

The efficiency of succinic acid production from lignocellulosic biomass by fermentation with *C. glutamicum* is affected by various factors, including the cell concentration, *k*_L_a, anaerobic conditions and optimal glucose concentration (*11*, *12*). Under semi-anaerobic conditions, with a cell dry mass of 10 g/L and a *k*_L_a value of 9.6 h^-1^, fermentations using *ΔldhA-L6* were conducted on 1 % hydrolysates derived from different lignocellulosic biomass (in g/L: pine 8.93, poplar 9.27, oak 8.44, bamboo 9.93, and spent coffee grounds 8.52) to analyse the effects of the sugar composition on succinic acid productivity under unsaturated sugar conditions (Fig. 3). The glucose in the hydrolysates of poplar, oak, bamboo and spent coffee grounds was depleted within 3 to 6 h and converted into succinic acid. The productivity at the initial stage of each hydrolysate showed an efficient conversion rate to succinic acid in the following order: poplar>oak=bamboo>>pine>spent coffee grounds. Under saturated conditions with 5 % hydrolysates, the patterns observed in the hydrolysates of poplar and spent coffee grounds were opposite of those seen in the previous results: oak=spent coffee grounds≥bamboo>poplar=pine. The by-products, lactic acid and acetic acid, were first detected at the 24-hour fermentation checkpoint with 5 % hydrolysate. Lactic acid was primarily observed during the fermentation of pine and bamboo hydrolysates. Although produced in small amounts, acetic acid appeared to be generated in proportion to the pattern of succinic acid production. It seems that the production of these by-products does not affect succinic acid metabolism. Based on the results in Fig. 3g, succinic acid production was the highest in oak hydrolysate, followed by hydrolysates of bamboo and poplar. The optimal amount of hydrolysate for succinic acid production was determined to be 4 %, considering economic factors such as electricity, enzyme dosage and sugar utilization efficiency. Although the consumption rate of xylose was much slower than of glucose, a comparison of the results of the two hardwoods and the bamboo (all of which contain xylose-rich hemicellulose) with those of the softwood and spent coffee grounds showed no correlation between xylose and succinic acid fermentation. Next, we investigated which types of derivatives or monosaccharides affect succinic acid production during fermentation with *C. glutamicum*.

### Effects of monosaccharides on succinic acid production

**Fig. 4:**
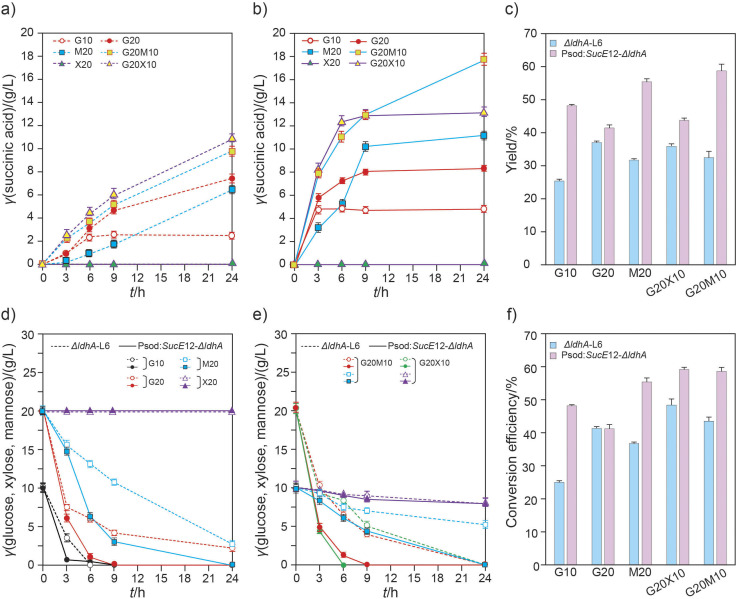
Succinic acid production using the engineered *Corynebacterium glutamicum* strains on monosaccharides. Succinic acid production by: a) *ΔldhA-L6* and b) Psod:*SucE*12-*ΔldhA* was observed during 24 h of fermentation of monosaccharides and their combinations. Dotted line: *ΔldhA-L6*, solid line: Psod:*SucE*12-*ΔldhA*, c) the yield after 24 h of fermentation using monosaccharides, d) and e) monosaccharide consumption when using the sole monosaccharide and two-monosaccharide combinations, respectively. Dotted line: *ΔldhA-L6*, solid line: Psod:*SucE*12-*ΔldhA*, circle: glucose, square: mannose, triangle: xylose, f) the conversion efficiency after 24 h of fermentation using monosaccharides. G10 and G20=10 and 20 g/L glucose, respectively; X5, X10 and X20=5, 10 and 20 g/L xylose, respectively; M5, M10, and M20=5, 10 and 20 g/L mannose, respectively

To demonstrate the correlation between monosaccharides and succinic acid fermentation, two transgenic strains, *ΔldhA-L6* and Psod:*SucE12-ΔldhA*, were used to ferment different combinations of pure monosaccharides, such as glucose, xylose and mannose (Fig. 4). During the initial stages (3−6 h) of G10 and G20 fermentation, the succinic acid transporter overexpressing strain, Psod:*SucE12-ΔldhA*, showed succinic acid conversion rates that were approx. 2.01−4.91 and 1.20−1.49 times higher than that in *ΔldhA-L6* strain, lasting approx. 1.91 and 1.72 times longer than 24 h, respectively (Fig. 4). The Psod:*SucE12-ΔldhA* strain showed much higher succinic acid production than the *ΔldhA-L6* strain in the sole presence of mannose (M20). Notably, when mannose was used in combination with glucose (G20M10), the Psod:*SucE12-ΔldhA* strain remarkably improved succinic acid production during the initial stages (approx. 2.54−3.38 times higher production than that with *ΔldhA-L6* strain). Additionally, the increase was 1.32−1.61 times higher than that observed with glucose alone (G20) in the same strain, Psod:*SucE12-ΔldhA*, persisting approx. 2.12 times longer than 24 h, demonstrating theoretical succinic acid productivity similar to that obtained with 40 g/L glucose. The succinic acid fermentation of xylose with glucose also showed patterns similar to those observed with mannose. All values related to the consumption and conversion of glucose, xylose and mannose to succinic acid were much higher in the Psod:*SucE12-ΔldhA* strain than in the *ΔldhA-L6* strain (Fig. 4c and Fig. 4f). Overall, the results indicated that the succinic acid transporter and the monosaccharides xylose and mannose have a positive impact on succinic acid production when combined with glucose.

**Fig. 5:**
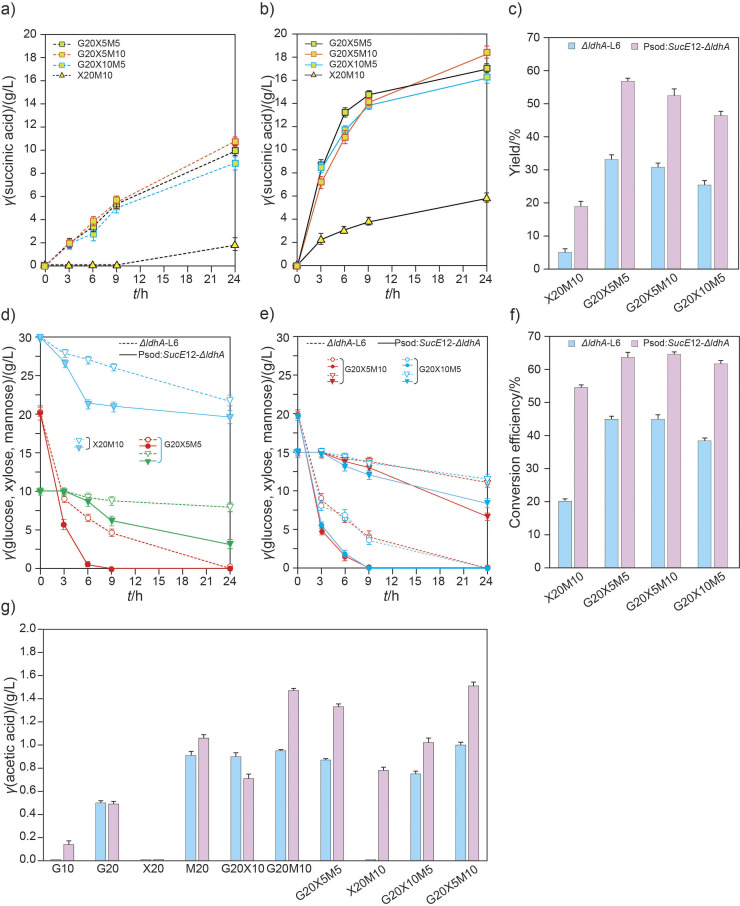
Comparison of succinic acid production and monosaccharide consumption by the engineered *Corynebacterium glutamicum* strains: a) succinic acid production by *ΔldhA-L6* and b) Psod:*SucE*12-*ΔldhA* was observed during 24 h of fermentation of the three-monosaccharide combinations. Dotted line: *ΔldhA-L6*, solid line: Psod:*SucE*12-*ΔldhA*, c) the yield of the three-monosaccharide combination, d and e) monosaccharide consumption when using different monosaccharide combinations. Dotted line: *ΔldhA-L6*, solid line: Psod:*SucE*12-*ΔldhA*, circle: glucose, reversed triangle: xylose+mannose, f) the conversion efficiency of the three-monosaccharide combination, g) acetic acid was more actively produced using different mixtures of sugars

The triple combinations of glucose, xylose and mannose (G20X5M5, G20X10M5 and G20X5M10) resulted in 2.05−2.21 times higher succinic acid production during 24 h than that of G20 in the Psod:*SucE12-ΔldhA* strain, with the glucose in these triple combinations being completely consumed by 9 h (Fig. 5). Lactic acid was not detected in any of the combinations, but the acetic acid concentrations were remarkably higher when the metabolism of succinic acid production was more active.

Succinic acid production significantly increased in the Psod:*SucE12-ΔldhA* strain compared to the *ΔldhA-L6* strain. Thus, the increased metabolism of succinic acid production by the transporter and monosaccharides was corroborated.

### Optimization of succinic acid production from woody and herbaceous plants

Succinic acid production using *C. glutamicum*-derived strains and hydrolysates from lignocellulosic biomass as a carbon source has not yet been reported. In our analysis of succinic acid production using hydrolysates obtained from woody and herbaceous plants, we observed that a higher concentration of hydrolysate does not lead to a proportional increase in succinic acid production (Fig. 3). Additionally, we investigated the effect of monosaccharides (glucose, xylose and mannose) on the rate of succinic acid conversion by the recombinant strains. We observed a positive tendency towards succinic acid production, except in two cases involving the absence of glucose or the sole presence of xylose. Although a small amount of xylose was consumed in some cases, it did not disturb glucose consumption and conversion to succinic acid in either strain. However, the factor limiting the hydrolysate content during the fermentation process remains unknown. To address this issue, we initially focused on glucose and explored the effects of concentrations ranging from 20 to 50 g/L during fermentation with the Psod:*SucE*-*ΔldhA* strain. The optimal glucose concentration was determined to be 30 g/L, as it showed the most favourable balance between glucose consumption and succinic acid production and served as a limiting factor when concentration exceeded 30 g/L (Fig. S2).

**Fig. 6:**
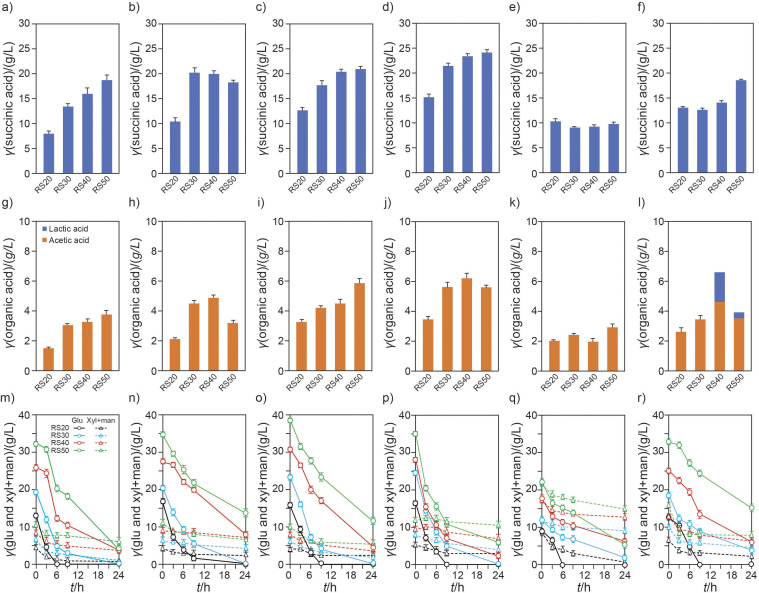
Succinic acid production from woody and herbaceous plants using Psod:*SucE*12-*ΔldhA* strain: succinic acid production using hydrolysates from: a) pine, b) poplar, c) oak, d) bamboo, e) spent coffee grounds, and f) a mixture was analysed after 24 hours of fermentation. The production of organic acids, including acetic and lactic acids, using hydrolysates from: g) pine, h) poplar, i) oak, j) bamboo, k) spent coffee grounds, and l) a mixture was analysed after 24 hours of fermentation. No lactic acid was detected during 24 h of fermentation. Sugar consumption using hydrolysates from: m) pine, n) poplar, o) oak, p) bamboo, q) spent coffee grounds, and r) a mixture was assessed after 24 hours of fermentation. All data were based on the results of the 24-hour fermentation. Glu=glucose, Xyl=xylose, Man=mannose

In the enzymatic saccharification of lignocellulosic biomass, variations in the final concentration of reducing sugars are often observed. These variations can be attributed to different factors, including pretreatment and substrate conditions, enzyme amount and reactions, and the method used for reducing sugar measurement. To improve the accuracy of determining the final concentration of reducing sugars, reducing sugar sets (RS20, RS30, RS40 and RS50, representing the concentration in g/L) derived from pine, poplar, oak, bamboo, spent coffee grounds and their mixture were prepared and used to estimate the efficiency of succinic acid production with Psod:*SucE*12*-ΔldhA* (Fig. 6). The composition of glucose and xylose with mannose in RS20−RS50 remained consistent, accounting for approx. 75 and 24 % in pine, 77 and 22.73 % in poplar, 80.05 and 19.95 % in oak, 75.56 and 24.44 % in bamboo, 50.81 and 49.19 % in spent coffee grounds and 73.02 and 26.98 % in the mixture, respectively. However, the residues that remained after the enzymatic saccharification of the woody and herbaceous substrates caused changes in the sugar compositions compared to the data in Table 1. It can be assumed that the highly compact and recalcitrant cellulose chains in pine, poplar, oak and bamboo plants contributed to the changes in glucose concentration (*10*).

A substantially high amount of unhydrolyzed residues remained after the enzymatic hydrolysis of spent coffee grounds. These residues were inferred to contain mannan polymer, lipophilic fractions, ethanol- and water-soluble compounds and compounds soluble in 1 % NaOH (*31*). In this study, a mannanase, Man5A, was identified in the cellulase cocktail solution from *Trichoderma reesei* (*32*). The small amount of Man5A in the cellulase cocktail may contribute to the relatively lower release of mannose from spent coffee grounds.

During the fermentation of reducing sugars derived from woody plants, hardwood exhibited a more favourable tendency to succinic acid production than softwood (Fig. 6). The optimal concentrations of the hydrolysate for succinic acid production were found to be RS50 for pine, RS30 for poplar and RS40 for oak. The hemicellulose in pine wood contains six times higher amounts of mannose than that in hardwood, with a xylose to mannose ratio of approx. 1:2. Based on the results from G20X5M10 in Fig. 5, the monosaccharide composition of pine hydrolysate is theoretically more conducive to succinic acid production. Additionally, the side pathway for acetic acid is more pronounced during the fermentation of hardwood than that of softwood, implying that more acetyl-CoA is used for acetic acid production during hardwood fermentation. Despite these factors, the succinic acid production from pine wood (13.45−18.80 g/L succinic acid for 24 h) was lower than that from xylose-rich hardwoods (17.69−21.09 g/L succinic acid for 24 h) and bamboo (21.41−24.12 g/L succinic acid for 24 h).

Bamboo, among herbaceous plants, has a higher cellulose content than softwood and hardwood (Table 1). Moreover, its unique microfibril structure, comprising lower lignin content (10 %) and hemicellulose with higher xylose content, is advantageous during pretreatment and enzymatic hydrolysis (*21*). These properties make bamboo a valuable bioresource for the production of bioenergy and biochemicals. The xylose-to-mannose ratio of HPAC-pretreated bamboo was determined to be 15:1 (Table 1) and the hydrolysate contained 75.56 % glucose and 24.44 % hemicellulose derivatives. Thus, bamboo hydrolysate is more abundant in xylose than the other studied materials. When applied to succinic acid fermentation, 21.4−24.12 g/L succinic acid was produced from the RS30−RS50 bamboo hydrolysates over 24 h (Fig. 6). Among the lignocellulosic biomass examined in this study, bamboo hydrolysates produced the highest amount of succinic acid. Furthermore, succinic acid production from the RS20 bamboo hydrolysate was comparable to that from the RS40 pine hydrolysate.

Spent coffee grounds have much higher lignin and mannan-based hemicellulose contents than pine, oak, poplar and bamboo (Table 1). However, their lower cellulose content, which indicates a limited glucose supply, is a disadvantage for the bioconversion process. However, spent coffee grounds are useful for analysing and comparing the patterns of succinic acid production in lignocellulosic biomass due to their high mannose content. After enzymatic hydrolysis, the glucose-to-mannose ratio in the hydrolysate changed from 1:1.8 to 1:1. However, despite the much higher mannose content in the hydrolysate, the succinic acid production in spent coffee grounds was observed to be the lowest among the woody and herbaceous plants (Fig. 6). Compared to the data in Fig. 3, the overexpression strain Psod:*SucE-ΔldhA* showed a 36.27 % decrease in succinic acid production at 4−5 % and RS40−RS50 compared to the *ΔldhA* strain. Similar patterns were observed during the fermentation of the mixture RS20−RS50.

In the case of fermentation of mixed samples, the acetic acid-to-succinic acid ratio was much higher in the mixture (0.38−0.53) than in bamboo (0.23−0.27), indicating that acetic acid production is more active in the fermentation of the mixture. In particular, lactic acid was first observed at 24 h during the fermentation of the mixture.

Lignocellulosic biomass serves as a sustainable carbon source for alternative energy and chemical production. Pretreatment with HPAC is efficient in removing lignin and promoting the swelling of cellulose fibres in the microfibril structure of lignocellulosic biomass. Thus, it facilitates efficient enzymatic hydrolysis, while cellulose is a supplier of glucose and hemicellulose supplies a diverse range of sugars, including xylose, mannose, arabinose and galactose. Xylose and mannose, derived from hemicellulose, contribute to the high efficiency of glucose−succinic acid metabolism in the Psod:*SucE-ΔldhA* strain. However, these derivatives tend to be consumed more slowly than glucose during succinic acid fermentation, resulting in a higher residual proportion of xylose and mannose. In addition, acetic acid is a major by-product, with its production pattern mirroring that of succinic acid from hydrolysates. The genes acetate kinase (ackA) and acetyl-CoA synthase (acsA), which influence acetic acid metabolism during fermentation, have been engineered to improve succinic acid production. It is evident that further metabolic engineering of xylose and acetic acid conversion to succinic acid is the next challenge for achieving even more efficient succinic acid production from poplar, oak and bamboo.

## CONCLUSIONS

The engineered *Corynebacterium glutamicum* strains were used for the production of succinic acid from different combinations of monosaccharides and lignocellulosic biomass. The Psod:*SucE*-*ΔldhA* strain increased the succinic acid production by 12−91 % compared to the *ΔldhA* strain when using pure monosaccharide sources and their combinations. Notably, mannose was found to induce efficient succinic acid production. Among the lignocellulosic biomass, bamboo proved to be an optimal carbon source for succinic acid production, followed by hardwood (oak and poplar), softwood and spent coffee grounds.

## Appendix

**Table S1:** Primers to generate recombinant *Corynebacterium glutamicum* using CRISPR-cpf1 gene editing vector

pJYS3Am MCS	**Fragment 1 (Cpf1 gene)** Sense: 5’ CTTTAGGTCTCCCATGGTCATGACCTGAGCTGTTTACAATT 3’ Anti-sense: 5 GGTTCGTTCTCATGGCTCACGC 3’ **Fragment 2 (Rep101+ pBL1^st^)** Sense: 5’ CCTGCAGGCATGCAAGCTTAAGAG 3’ Anti-sense: 5’ GAAATGGTCTCCCATGGTGACCGCCTCGATGATCGCC 3’ **Fragment 3 (Amp gene)** Sense: 5’ CTTTAGGTCTCCCATGGAAAGGGCCTCGTGATAC 3’ Anti-sen: 5’ CCAATTCCACACATGGTACCACACGATGATTAATTGTAAACAGCTCAGGTCATGCTGA CAGTTA CCAATGCTTAATCAGTG 3’
Target DNA 1 set (crRNA + target DNA)	Sense: 5’ CCTGCAGGCATGCAAGCTTAAGAG 3’ Anti-sense: 5’ GAAATGGTCTCGGATCCGAATTTCTACTGTTGTAGATGAGTTGCATACGCAT ACGCACTGCAATTTAAATAAAACGAAAGGCTCAGTCG 3’
Target DNA 2 set (crRNA + target DNA)	Sense: 5’ GAAATGGTCTCACTAGTTTGACAGCTAGCTCAGTCCTAGGTATAATTCTAGAGAATTTC TACTGTTGTAGATGCGTCGATAATG 3’ Anti-sense: 5’ CTTTAGGTCTCGGGCCCATCGGCATTTTCTTTTGCGTTTCCCGGGCGCTGCCTATCA CATTATCGACGCATCTACAACAG 3’ Experiment: After annealing two primers, the short DNA fragments were synthesized. The PCR products were digested with restriction enzymes and inserted into the pJYS3Am_MCS vector.
pCold 1 vector	Construction of homologous arms and kanamycin selection marker gene.
*LdhA*p (Left arm)	Sense: 5’ GAAATGGTCTCGGGCCCGCTCCGGCTCCCACGGCTGCTC 3’ Anti-sense: 5’ GAAATGGTCTCGGTACCTTTCGATCCCACTTCCTGATT 3’
*LdhA* (Right arm)	Sense: 5’ CTTTAGGTCTCAAGCTTATCACCTTGCGATCATCGACAT 3’ Anti-sense: 5’ CTTTAGGTCTCACTAGTGTTGGCAGCGCAAGTGTTCCA 3’
Kn^r^ gene set (Promoter + gene + terminator)	Sense: 5’ CTTTAGGTCTCGGTACCGGAAGCGGAACACGTAGAAAGC 3’ Anti-sense: 5’ GAAATGGTCTCCTCGAGGGTCATGATTCCGCGAACCCA 3’
*LdhA*p (-1042)	Sense: 5’ CCGACAACACTGCACGGCCCTGCGA 3’
*LdhA*p (-202)	Sense: 5’ CAATTCTGCAGGGCATAGGTTGG 3’
*LdhA* (+332)	Anti-sense: 5’ CGCAGAGGAGAGGCGTTGCTGCAGG 3’
Promoter sequence	**Amp^r^ promoter:** 5’ CGTCAGGTGGCACTTTTCGGGGAAATGTGCGCGGAACCCCTATTTGTTTATTTTTCTAAATACA TTCAAATATGTATCCGCTCATGAGACAATAACCCTGATAAATGCTTCAATAATATTGAAAAAGGAAGAGT 3’ **PlacM promoter:** 5’ TGAGCTGTTTACAATTAATCATCGTGTGGTACCATGTGTGGAATTGGAAAGGACTTGAACG 3’ **J23119 promoter:** 5’ TTATACCTAGGACTGAGCTAGCTGTCAA 3’ **Kn^r^ promoter:** 5’ GGAAGCGGAACACGTAGAAAGCCAGTCCGCAGAAACGGTGCTGACCCCGGATGAATGTCAG CTACTGGGCTATCTGGACAAGGGAAAACGCAAGCGCAAAGAGAAAGCAGGTAGCTTGCAGTGGGCTTACATGGCGATAGCTAGACTGGGCGGTTTTATGGACAGCAAGCGAACCGGAATTGCCAGCTGGGGCGCCCTCTGGTAAGGTTGGGAAGCCCTGCAAAGTAAACTGGATGGCTTTCTTGCCGCCAAGATCTGATGGCGCAGGGGATCAAGATCTGATCAAGAGACAGGATGAGGATCGTTTCG 3’ **Psod promoter:** 5’ TGCGGAAACCTACGAAAGGATTTT 3’
Terminator sequence	**rmB T1 terminator:** 5’ ATTTGTCCTACTCAGGAGAGCGTTCACCGACAAACAACAGATAAAACGAAAGGCCCAGTCTT TCGACTGAGCCTTTCGTTTTATTT 3’ **SacB T1 terminator:** 5’ AAACGCAAAAGAAAATGCCGAT 3’ **Kn^R^ terminator** 5’ GCGGGACTCTGGGGTTCGCGGAATCATGACC3’
*rpsL* gene	5’ ATGCCAACTATTCAGCAGCTGGTCCGTAAGGGCCGCCACGATAAGTCCGCCAAGGTGGCTACC GCGGCACTGAAGGGTTCCCCTCAGCGTCGTGGCGTATGCACCCGTGTGTACACCACCACCCCT**AAG**AAGCCTAACTCTGCTCTTCGTAAGGTCGCTCGTGTGCGCCTTACCTCCGGCATCGAGGTTTCCGCTTACATCCCTGGTGAGGGCCACAACCTGCAGGAGCACTCCATGGTGCTCGTTCGCGGTGGTCGTGTTAAGGACCTCCCAGGTGTCCGTTACAAGATCGTCCGTGGCGCACTGGATACCCAGGGTGTTAAGGACCGCAAGCAGGCTCGTTCCCG CTACGGCGCGAAGAGGGGATAA 3’ (Under line: point mutagenesis site, AAG →AGG)
Kanamycin resistance gene target DNA	5’ GAAATGGTCTCGGATCCGAATTTCTACTGTTGTAGATAACAAGATGGATTGCACGCAGGTTAA TTTAAATAAAACGAAAGGCTCAGTCG 3’
*SucE*	5’ CTTTAGGTCTCGAATTCATGAGCTTCCTTGTAGAAAATC 3’ 5’ GAAATGGTCTCAAGCTTGATAAGTAGGAACAACAACGTTTG 3’
